# Supplementary material for: Orthohantavirus Isolated in Reservoir Host Cells Displays Minimal Genetic Changes and Retains Wild-Type Infection Properties
Source: Viruses. 2020 Apr 17;12(4):457. doi: 10.3390/v12040457 (PMC7232471; doi:10.3390/v12040457)
Supplement: Supplementary file 1 [file viruses-12-00457-s001.pdf]

Supplementary figures

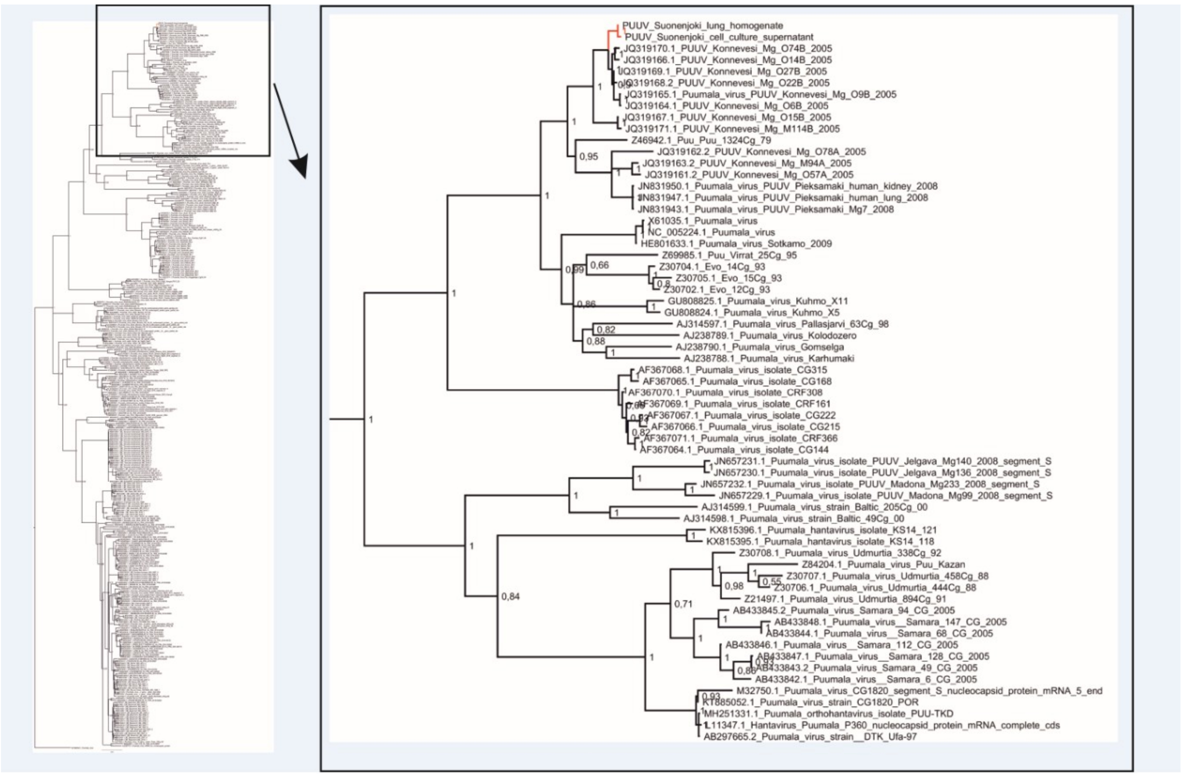

Figure S1. Phylogenetic analysis of the complete PUUV-Suo S segment. Analysis of both the original bank vole lung (lung homogenate) and Mygla.REC.B.-isolated (cell culture supernatant) are included and marked as red.

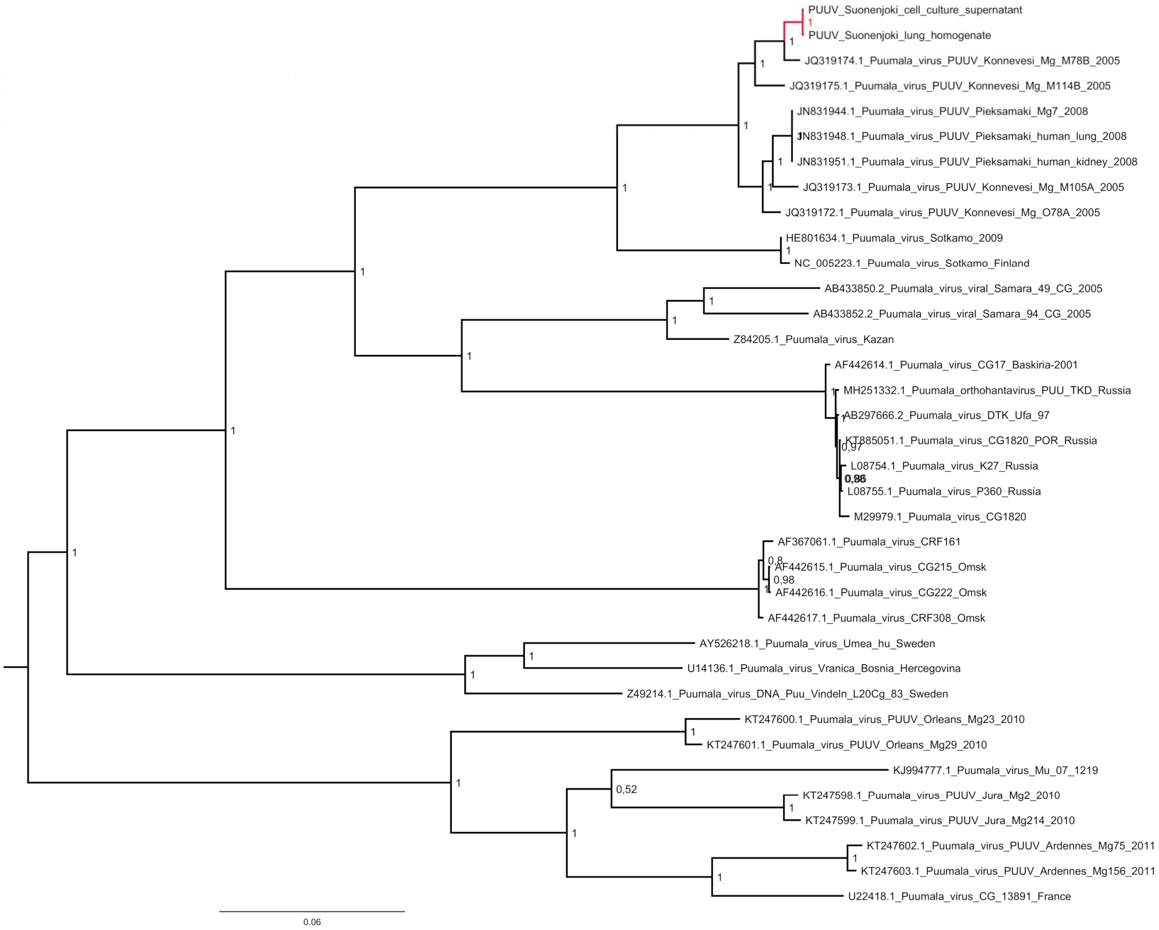

Figure S2. Phylogenetic analysis of the complete PUUV-Suo M segment. Analysis of both the original bank vole lung (lung homogenate) and Mygla.REC.B.-isolated (cell culture supernatant) are included and marked as red.

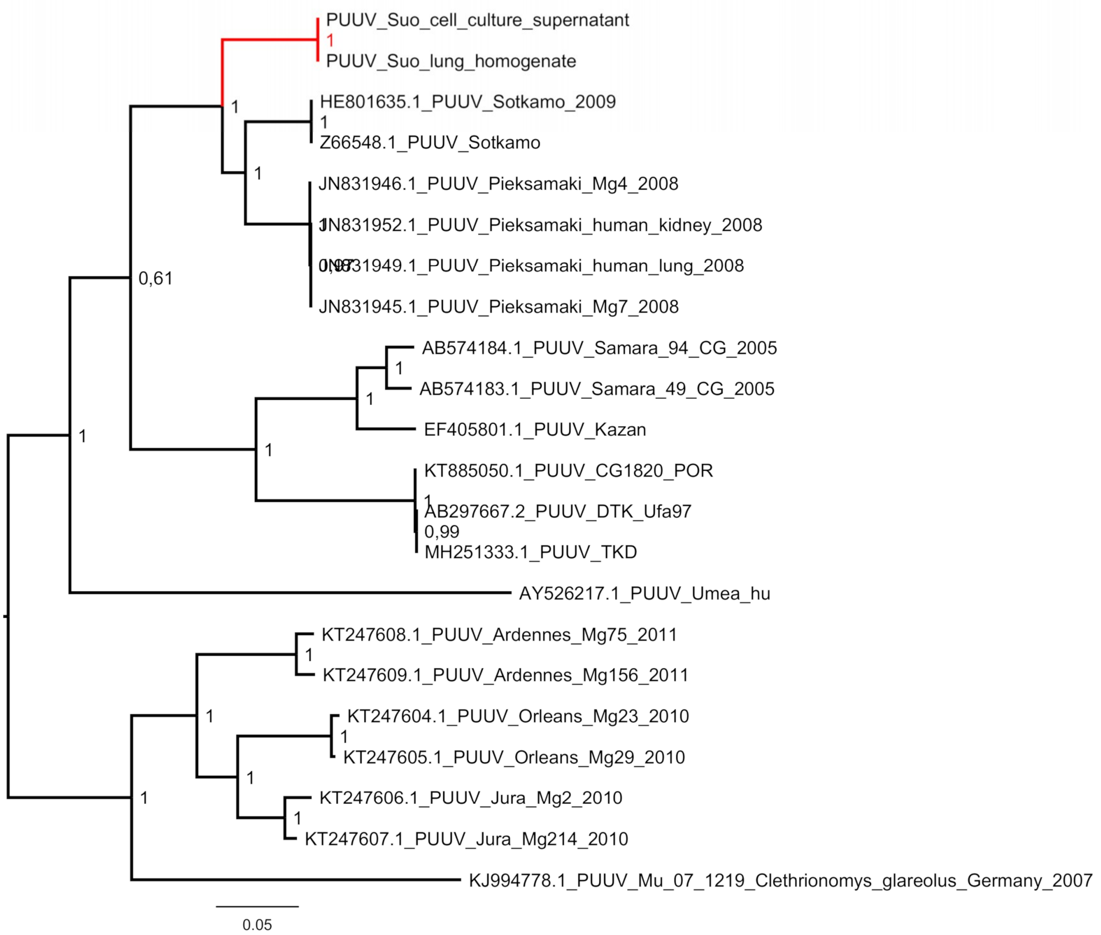

Figure S3. Phylogenetic analysis of the complete PUUV-Suo L segment. Analysis of both the original bank vole lung (lung homogenate) and Mygla.REC.B.-isolated (cell culture supernatant) are included and marked as red.

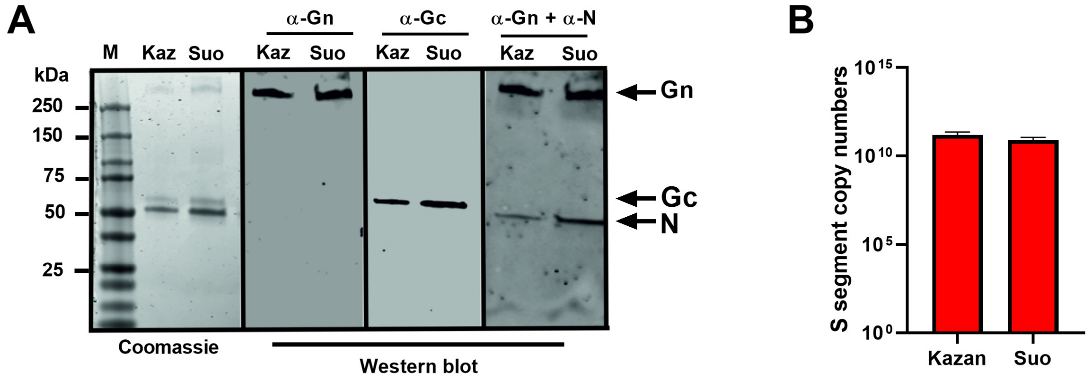

Figure S4. Comparison of viral protein and RNA levels in purified PUUV-Kazan and PUUV-Suo virus preparations. (A) Sucrose-cushion purified viruses were run into SDS-PAGE for Coomassie blue staining to reveal proteins separated by their molecular mass and western blot analysis to specifically detect viral glycoproteins Gn (>250 kDa, multimeric form of Gn), Gc (54 kDa) and nucleocapsid protein N (50 kDa). (B) PUUV S segment copy number quantification in purified PUUV-Kazan and PUUV-Suo virus stocks by qPCR.
